# Supplementary material for: SuperDendrix algorithm integrates genetic dependencies and genomic alterations across pathways and cancer types
Source: Cell Genom. 2022 Feb 9;2(2):100099. doi: 10.1016/j.xgen.2022.100099 (PMC8979493; doi:10.1016/j.xgen.2022.100099)
Supplement: Document S1. Figures S1–S16 [file mmc1.pdf]

Cell Genomics, Volume *2*

Supplemental information

SuperDendrix algorithm integrates genetic dependencies and genomic alterations across pathways and cancer types

Tae Yoon Park, Mark D.M. Leiserson, Gunnar W. Klau, and Benjamin J. Raphael

Supplementary Information

Supplementary Figures

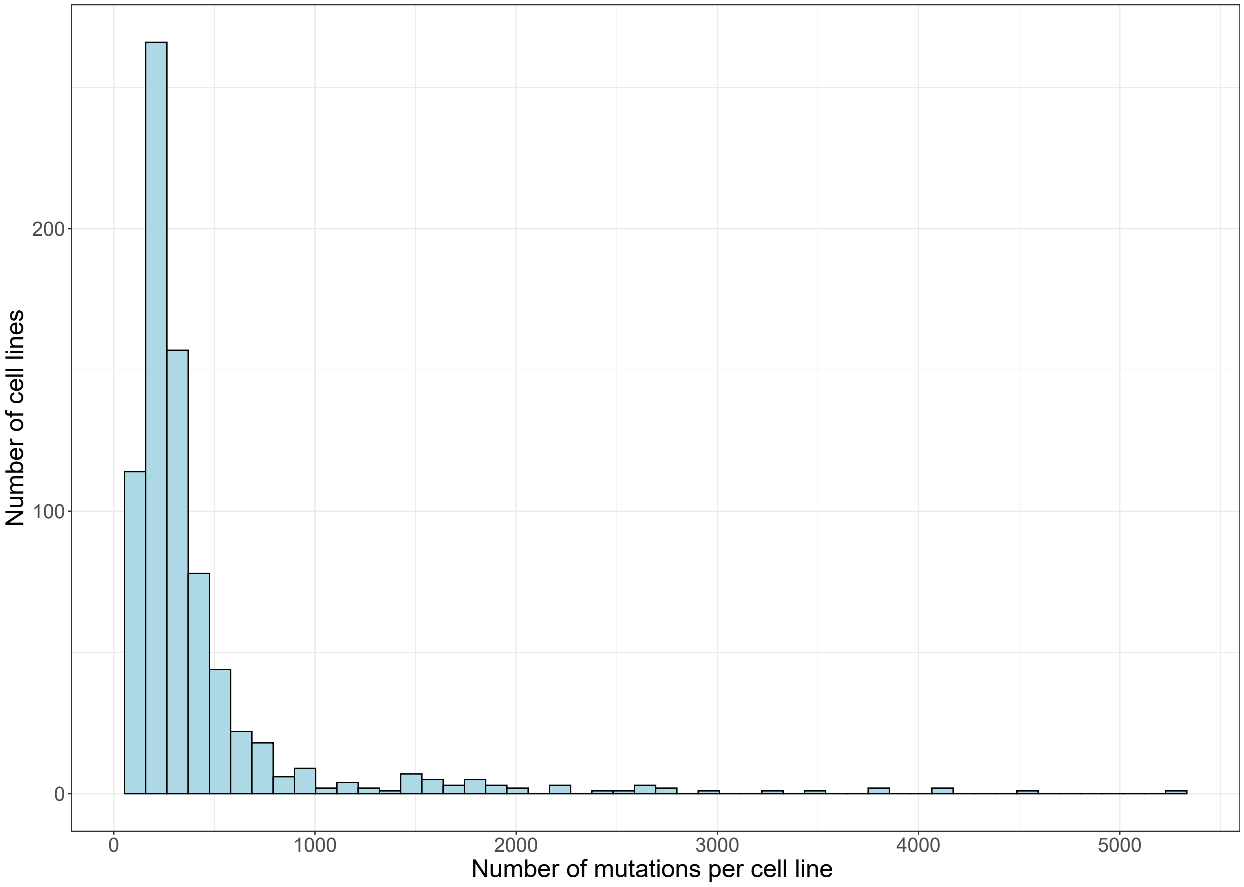

Figure S1: **Number of genomic alterations in cell lines in DepMap, Related to Figure 1.** Distribution of all non-synonymous mutations in the cell lines from DepMap. Mean = 410.01 and standard deviation = 533.43.

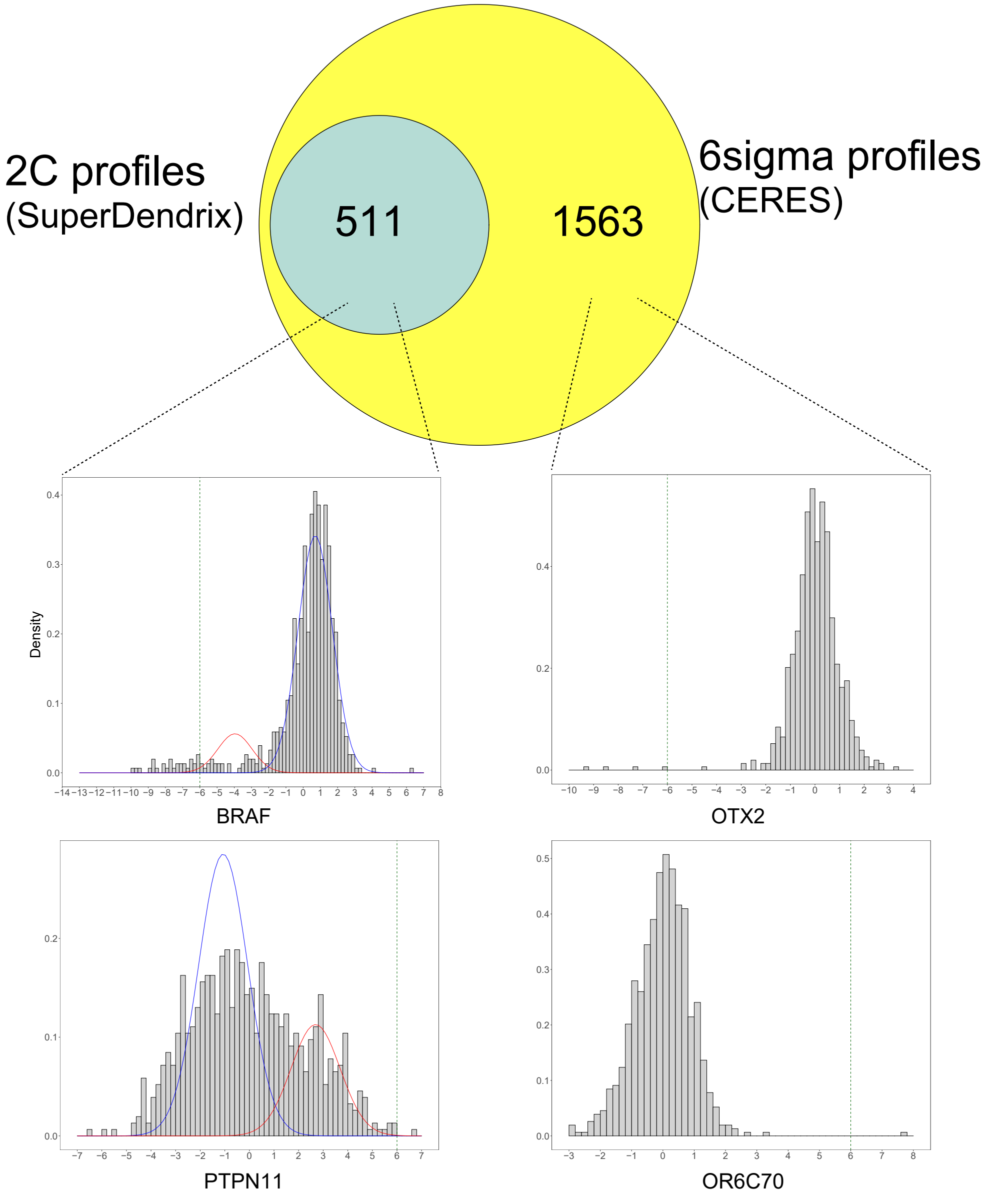

Figure S2: **Comparison of differential dependencies found by SuperDendrix and CERES, Related to Figure 2.** (Top) Overlap between differential dependencies identified using the two component mixture model in SuperDendrix and the 6σ threshold for CERES z-scores as described in Tsherniak et al.[15]. (Bottom) CERES z-score distributions for example profiles from each section of Venn diagram. The top row are profiles with increased dependency upon gene knockout, while the bottom row are profiles with decreased dependency. Red and blue curves indicate distributions in two component mixture, and green dashed lines represent 6σ thresholds for CERES z-scores.

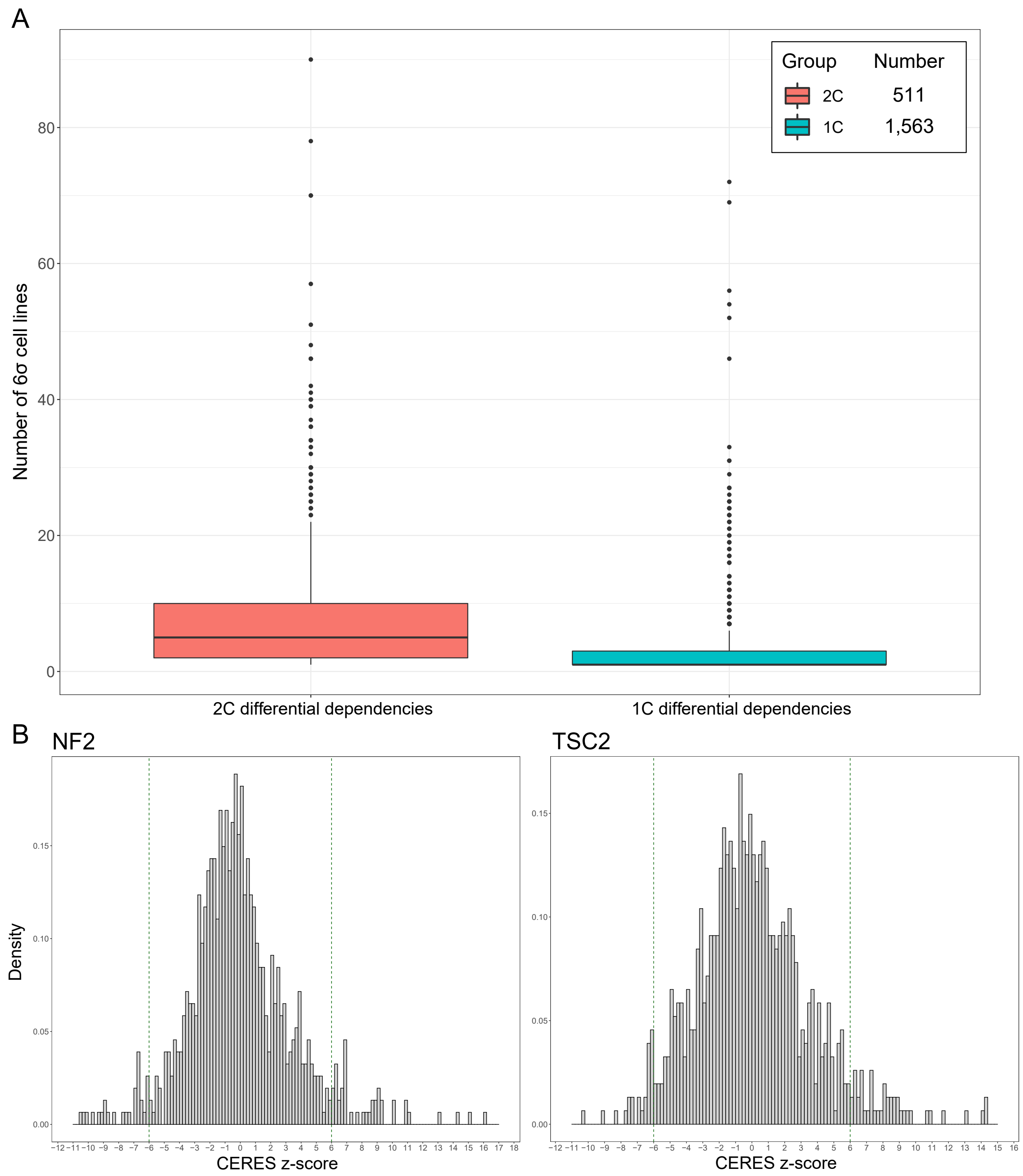

Figure S3: **Number of 6σ outlier cell lines in 2C and 1C profiles, Related to Figure 2.** (a) Box plots of number of cell lines with CERES scores $\geq 6\sigma$ from the mean for 2C profiles and 1C profiles. 2C profiles contain higher number of 6σ cell lines than 1C profiles on average (Welch t-test, $P < 0.001$). (b) CERES z-score distributions of *NF2* and *TSC2*, the 1C profiles containing the highest number of 6σ outlier cell lines (72 and 69). The outlier distributions have wide standard deviations: 3.47 and 3.52. The green dashed lines represent 6σ thresholds for CERES z-scores.

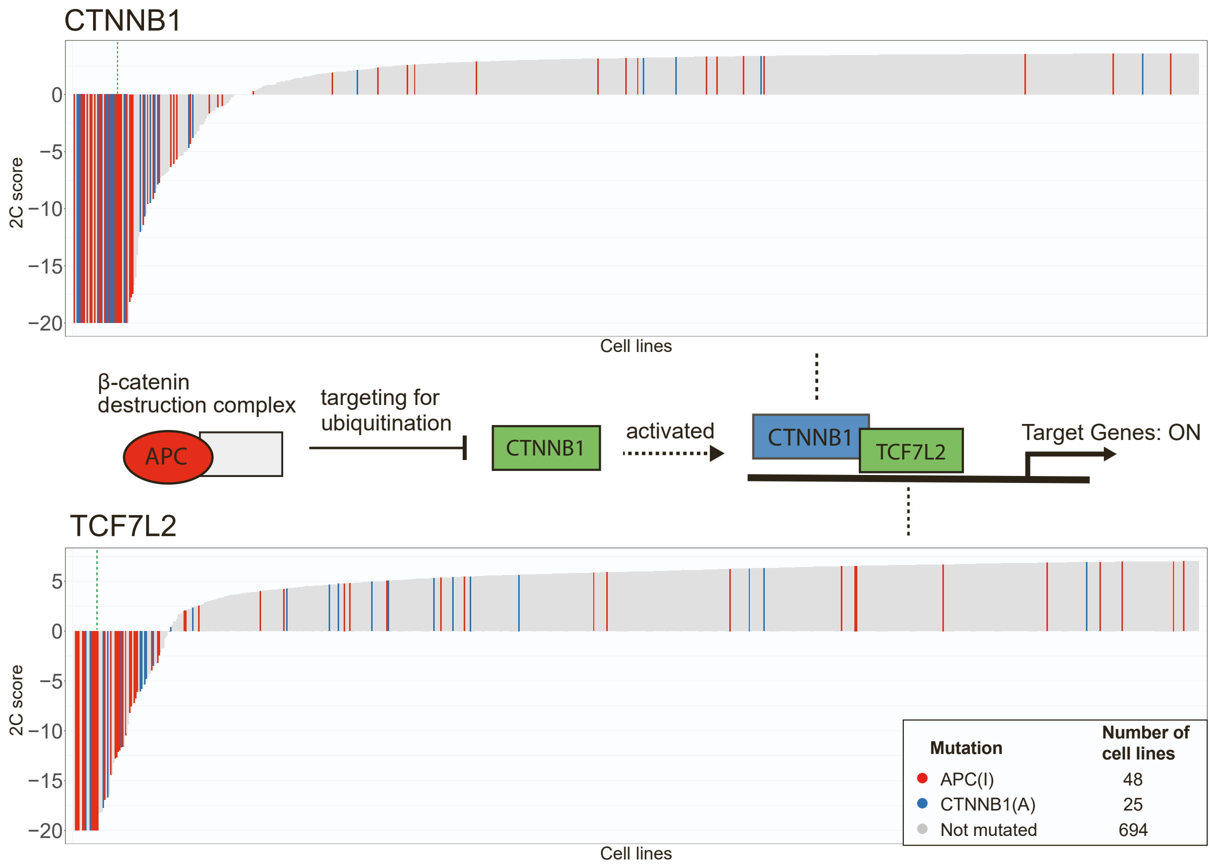

Figure S4: **SuperDendrix identifies dependencies in the Wnt pathway, Related to STAR Methods.** Cell lines with *APC* inactivating and *CTNNB1* activating mutations are associated with increased dependency on *CTNNB1* and *TCF7L2* (Same format as Figure 2B). Mutations in `APC(I)` and `CTNNB1(A)` are mutually exclusive as no cell line contains both mutations. Moreover, these dependencies are consistent with previously reported interactions in the Wnt pathway: when APC and correspondingly the β-catenin destruction complex are inactivated, CTNNB1 binds TCF7L2 in the nucleus and activates transcription of target genes[77].

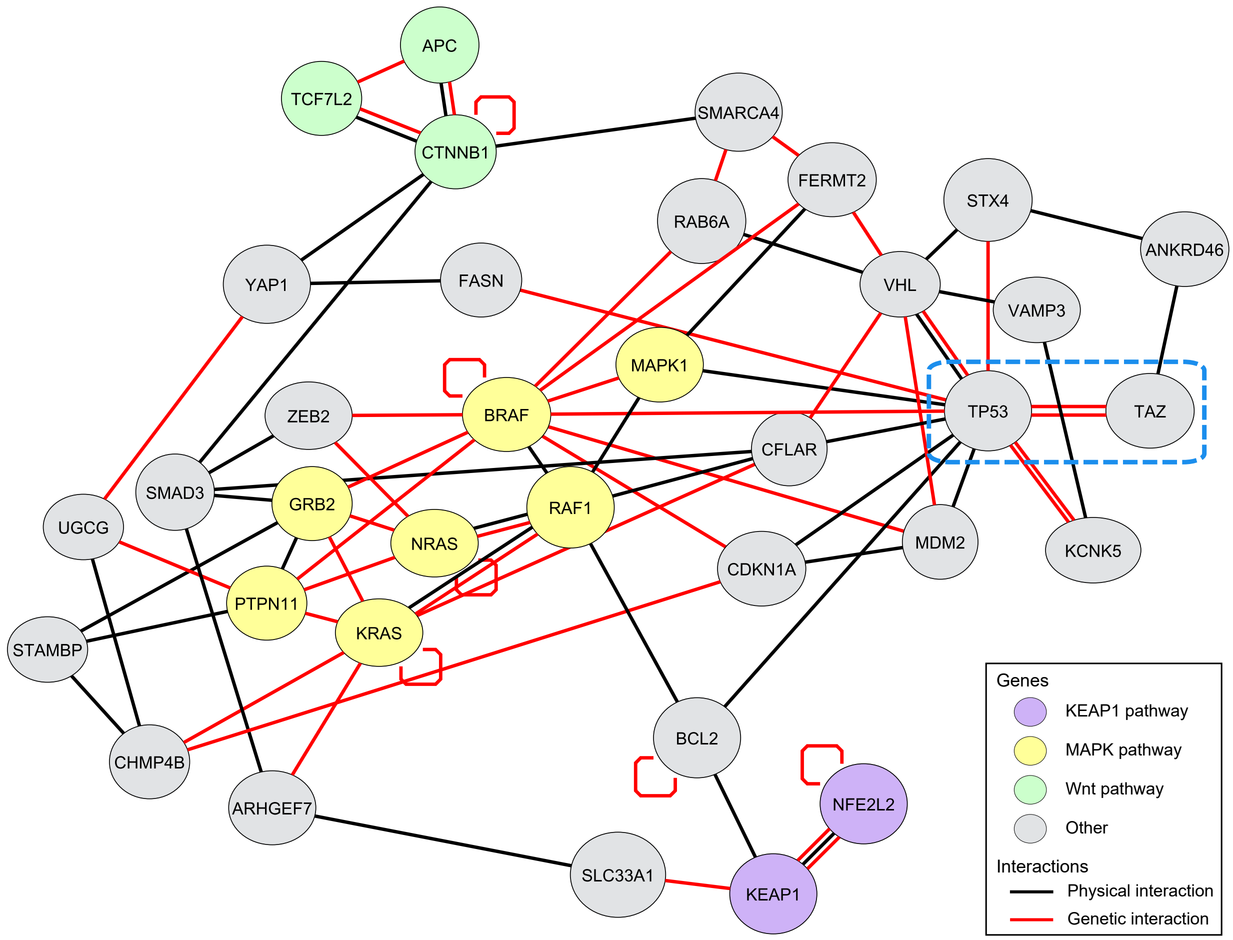

Figure S5: **The densest connected subnetwork of 35 nodes in a dual network containing physical interactions from the HINT+HI network and genetic interactions from associations reported by SuperDendrix, Related to STAR Methods.** The subnetwork contains genes across multiple pathways including the NFE2L2 pathway, the MAPK pathway, and the Wnt pathway as well as a novel association between TAZ dependency and mutations in TP53 that is not reported in the SuperDendrix analysis. The nodes represent differential dependencies or genes with mutations. Black edges correspond to physical interactions in the PPI network, and red edges correspond to associations between gene dependency and mutation identified by SuperDendrix.

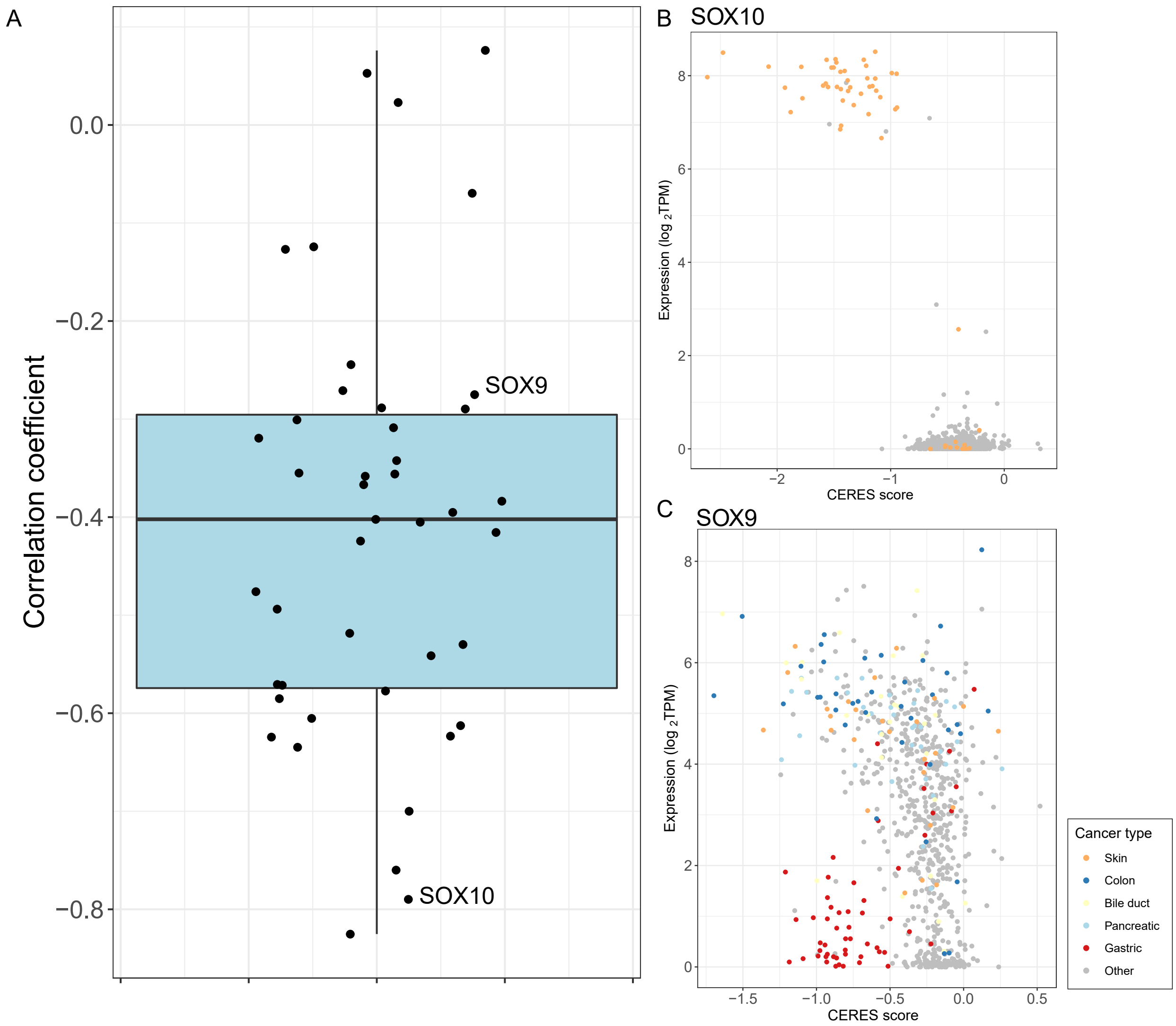

Figure S6: **Dependency on lineage-specific transcription factors is associated with both expression of the dependent gene and lineage classification, Related to Figure 5 and STAR Methods.** (A) Boxplot of Pearson correlation coefficients between expression of the dependent gene and its dependency scores across all cell lines. (B) *SOX10* expression vs. CERES dependency scores of *SOX10*. The majority of the cell lines with increased dependency on *SOX10* have elevated *SOX10* expression and are Skin cancer cell lines. (C) *SOX9* expression vs. CERES dependency scores of *SOX9*. Many of the dependent cell lines with high *SOX9* expression are from other cancer types. Also, Gastric cancer cell lines with increased dependency on *SOX9* have low *SOX9* expression.

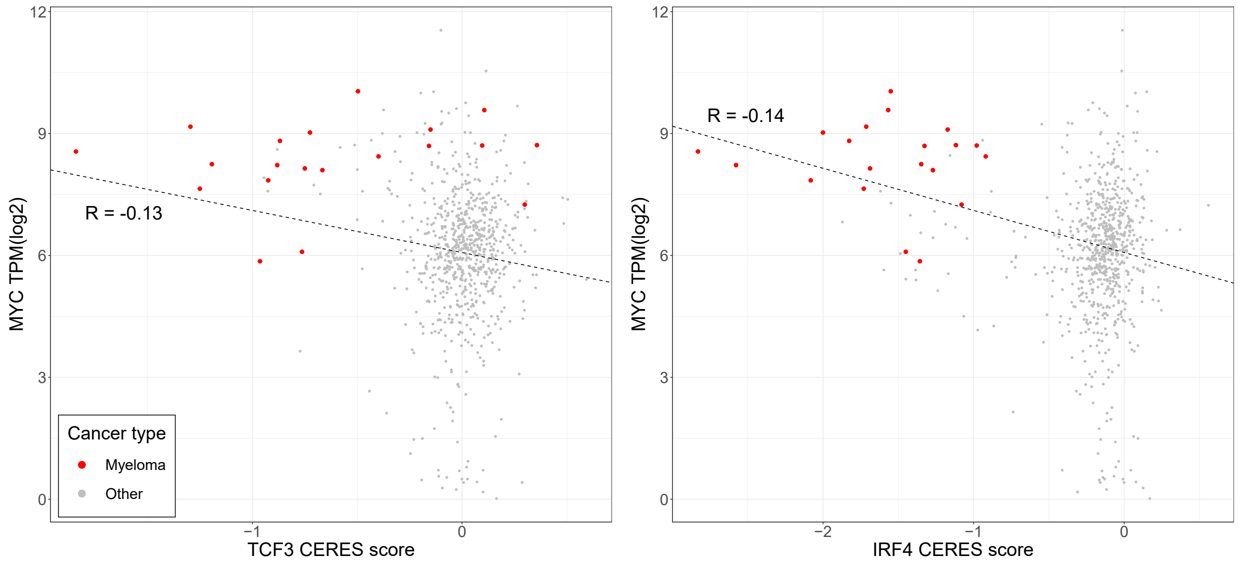

Figure S7: **Expression of *MYC* is correlated with *TCF3* and *IRF4* dependency, Related to Figure 6.** Correlations between expression of *MYC* and *TCF3* (left) and *IRF4* (right) dependencies are strong across all cell lines (*TCF3*: $R = -0.13$, $P \leq 0.001$, *IRF4*: $R = -0.14$, $P \leq 0.001$; Pearson correlation)

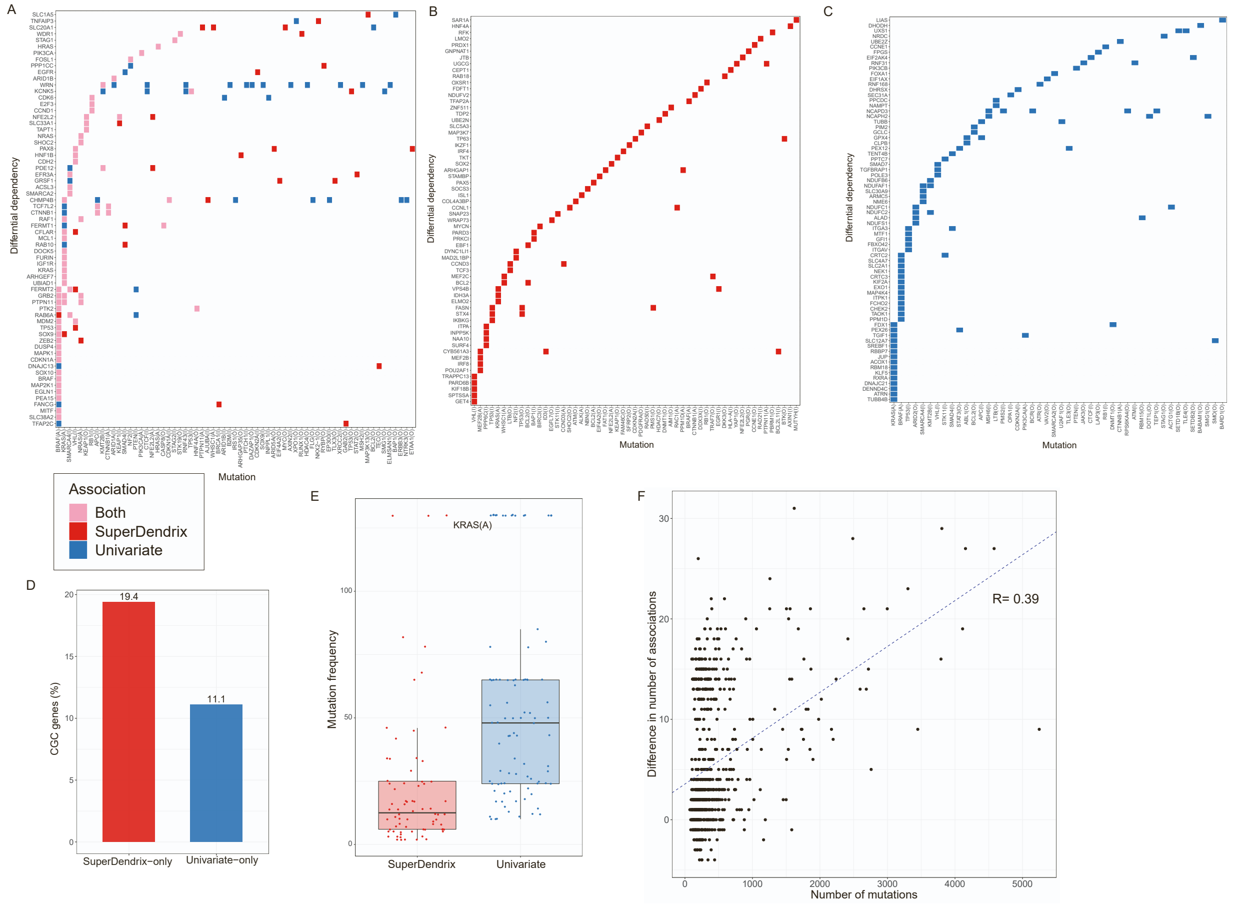

Figure S8: **Associations between differential dependencies (rows) and mutations (columns) identified by SuperDendrix and the univariate test, Related to STAR Methods.** (A) Differential dependencies that are reported by both methods to have associated mutations. (B) Associations reported uniquely by SuperDendrix. (C) Associations reported uniquely by the univariate test. (D-F) Associations identified by the univariate test contain fewer cancer genes and are biased towards frequent mutations and cell lines with a high number of mutations. (D) Percentage of differential dependencies reported uniquely by SuperDendrix or uniquely by the univariate test that are in the Cancer Gene Census (CGC) (E) Mutations in associations identified uniquely by the univariate test have higher frequency than those identified uniquely by SuperDendrix (univariate: 78.2, SuperDendrix: 41.6, $P = 0.011$; t-test). Mutation TP53(I) with outlier frequency (495 cell lines) was excluded from comparison. (F) The difference between the number of associations reported for a cell line by the univariate test and by SuperDendrix is positively correlated with the number of mutations in the cell line ($R = 0.39$, $P < 2.2e - 16$; Pearson correlation).

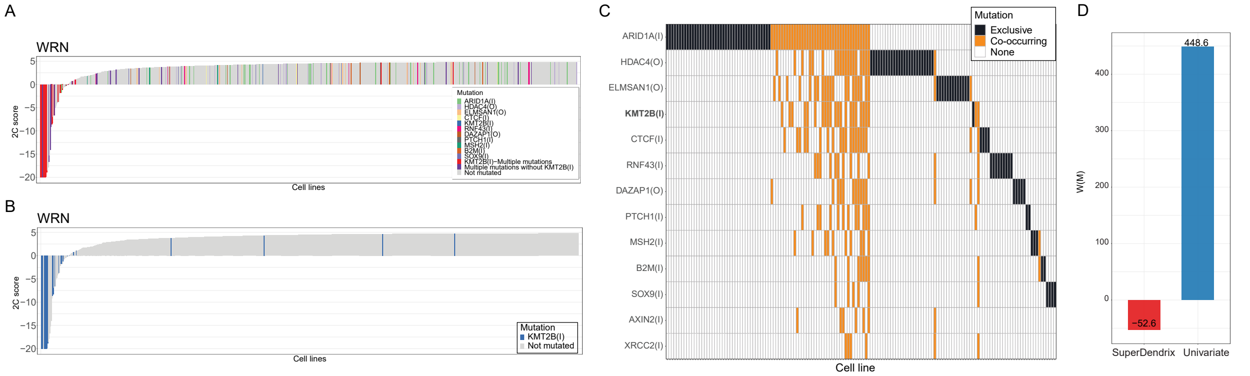

Figure S9: **Comparison of mutations reported by SuperDendrix and univariate test to be associated with increased dependency on *WRN*, Related to STAR Methods.** (A) Waterfall plot of *WRN* dependency scores colored according to presence of the 13 mutations reported by univariate test to be associated with *WRN* dependency. Two mutations, AXIN2(I) and XRCC2(I), that are not exclusive to any cell line are included in the "Multiple mutations" category. (B) Waterfall plot for the single mutation KMT2B(I) reported by SuperDendrix to be associated with *WRN* dependency shows that most of the cell lines with largest *WRN* dependency have KMT2B(I) mutations. (C) Mutation matrix for 13 mutations reported by univariate test and SuperDendrix to be associated with *WRN* dependency shows extensive co-occurrence between mutations. (D) SuperDendrix weight of KMT2B(I) mutation and set of 13 mutations.

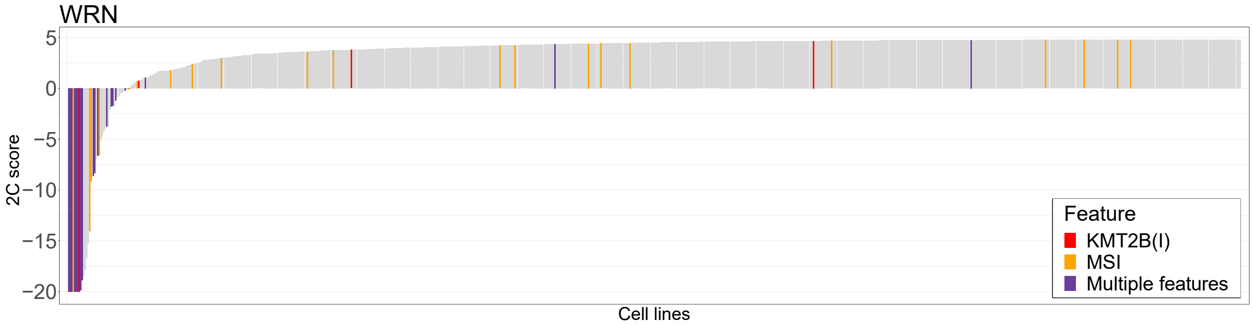

Figure S10: **Comparison of associations between increased dependency on *WRN* and KMT2B(I) mutation and MSI status, Related to STAR Methods.** Waterfall plot of *WRN* dependency for the mutation KMT2B(I) reported by SuperDendrix MSI status of cell lines shows extensive co-occurrence between the two features (20 of 24 cell lines containing KMT2B(I) mutations have MSI) as well as higher specificity of KMT2B(I) for increased dependency on *WRN* (Fraction of dependent cell lines: KMT2B(I): 18/24, MSI: 22/41).

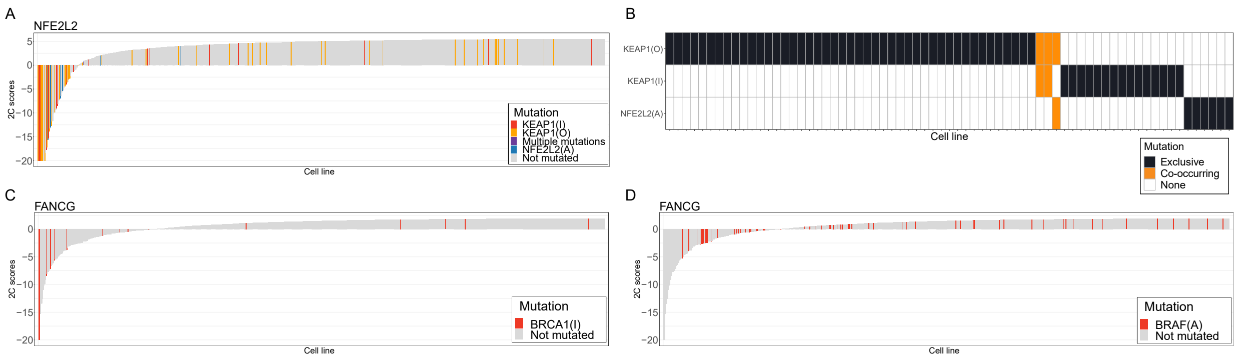

Figure S11: **Univariate test misses associations with rare mutations, Related to STAR Methods.** (A) Waterfall plot of *NFE2L2* dependency scores colored according to presence of the 3 mutations reported by SuperDendrix to be associated with *NFE2L2* dependency. The univariate test misses the association with a rare mutation, NFE2L2(A) (7 cell lines), and reports more frequent mutations only, KEAP1(I) (17 cell lines) and KEAP1(O) (48 cell lines). (B) Mutation matrix for 3 mutations reported by SuperDendrix to be associated with *NFE2L2* dependency shows approximate mutual exclusivity between mutations. (C) Waterfall plot of *FANCG* dependency scores colored according to presence of the single mutation BRCA1(I) (15 cell lines) reported by SuperDendrix to be associated with *FANCG* dependency. Most of the cell lines with the largest FANCG dependency have BRCA1(I) mutations. (D) Waterfall plot of the single mutation BRAF(A) (65 cell lines) reported by the univariate test to be associated with *FANCG* dependency shows that cell lines with largest *FANCG* dependency do not have BRAF(A) mutations.

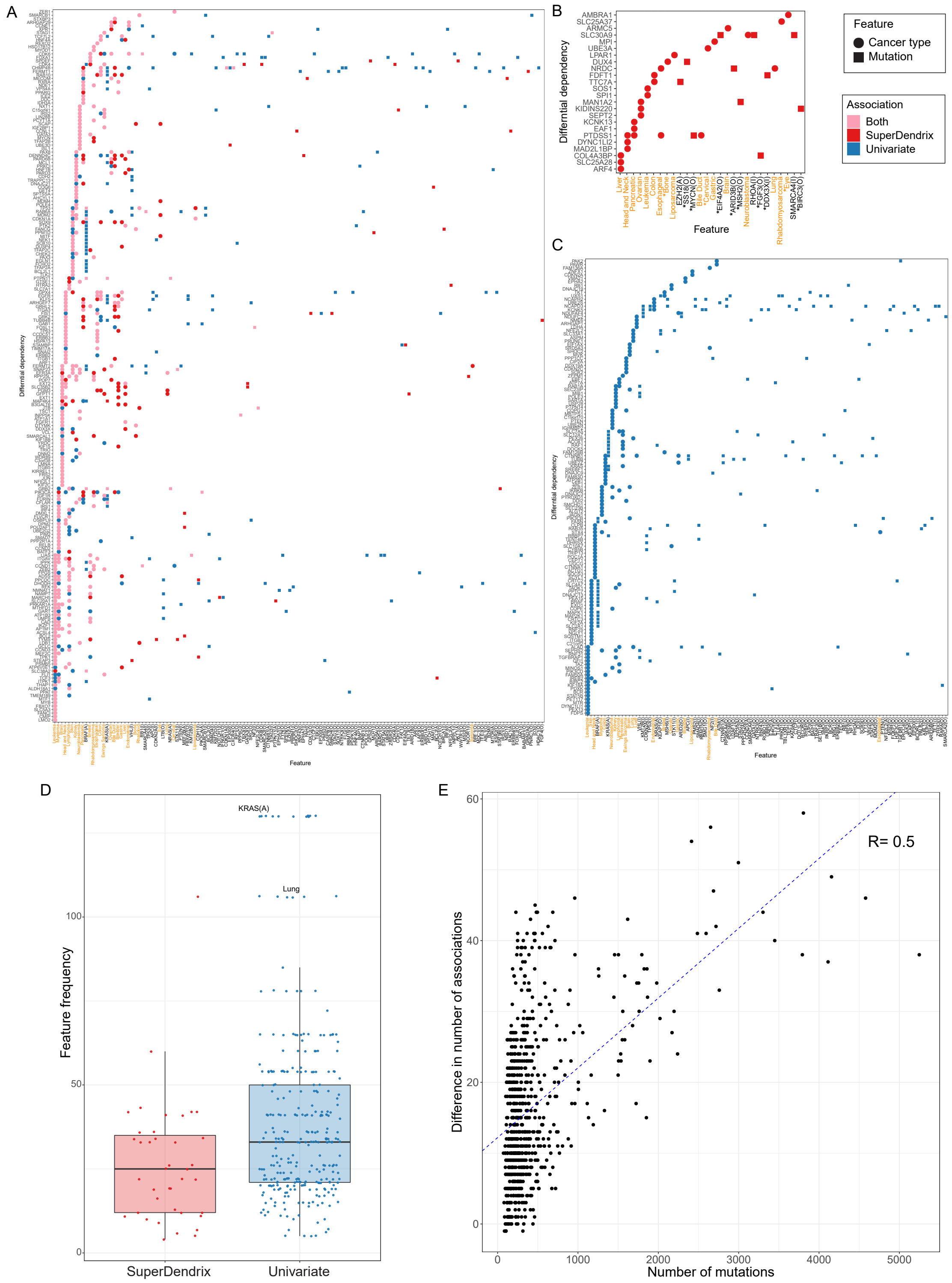
A
Differential dependency
Feature
B
AMBRA1
SLC25A37
ARMC5
SLC30A9
MPI
UBE3A
LPAR1
DUX4
NRDC
FDFT1
TTC7A
SOS1
SPI1
MAN1A2
KIDINS220
SEPT2
KCNK13
EAF1
PTDSS1
DYNC1LI2
MAD2L1BP
COL4A3BP
SLC25A28
ARF4
Differntial dependency
Liver
Head and Neck
Pancreatic
Ovarian
Leukemia
Colon
Esophageal
*Bone
Liposarcoma
EZH2(A)
*SS18(O)
*MYCN(O)
Bile Duct
*Cervical
Gastric
*EIF4A2(O)
Brain
*ARID3B(O)
*MSH2(O)
Neuroblastoma
RHOA(I)
*FGF3(O)
*DDX3X(I)
Lung
Rhabdomyosarcoma
*Eye
SMARCA4(I)
*BIRC3(O)
Feature
Feature
Cancer type
Mutation
Association
Both
SuperDendrix
Univariate
C
Differential dependency
Feature
D
KRAS(A)
Lung
Feature frequency
SuperDendrix
Univariate
E
R= 0.5
Difference in number of associations
Number of mutations

Figure S12: **Associations between differential dependencies (rows) and cancer types (columns labeled in gold) and/or mutations (columns labeled in black) identified by SuperDendrix and the univariate test, Related to STAR Methods.** (A) Associations for differential dependencies reported by both methods. (B) Associations reported uniquely by SuperDendrix. (C) Associations reported uniquely by the univariate test. (D-E) Associations reported uniquely by the univariate test are biased towards frequent features and cell lines with high number of mutations. (D) Cancer types and mutations in associations identified uniquely by the univariate test have higher frequency than those identified uniquely by SuperDendrix (univariate: 39.2, SuperDendrix: 26.7, $P = 0.002$; t-test). Mutation TP53(I) with outlier frequency (495 cell lines) was excluded from comparison. (E) The differences between the number of associations reported by the univariate test and SuperDendrix for each cell line is positively correlated with the number of mutations in the cell line ($R = 0.5$, $P < 2.2e - 16$; Pearson correlation).

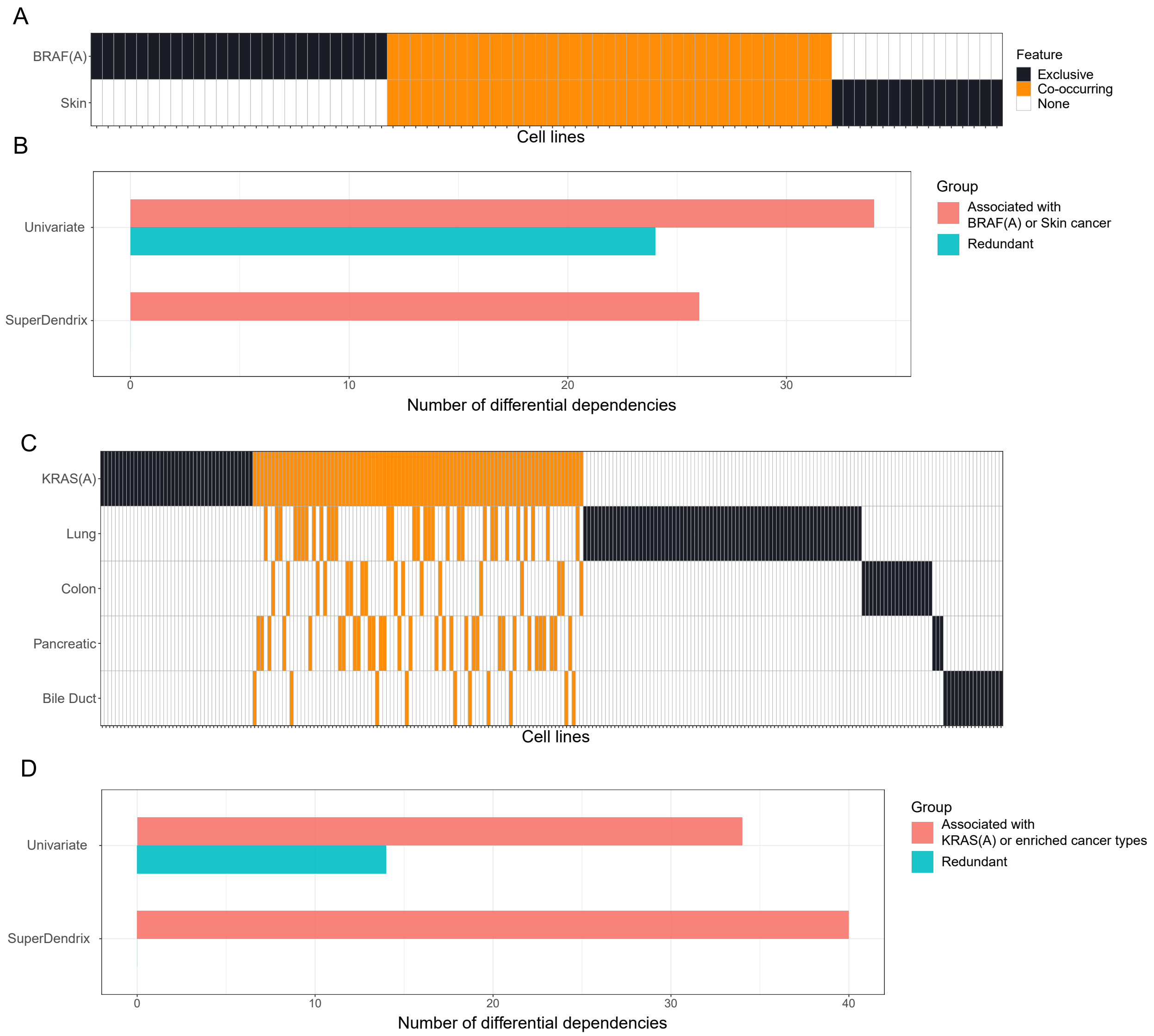

Figure S13: **The univariate test finds redundant associations between mutations and the cancer types that are enriched for these mutations, Related to STAR Methods.** (A) 65 cell lines with BRAF(A) mutation are significantly enriched for Skin cancer (39/65 cell lines with BRAF(A), fold-enrichment = 8.52, $P = 1.2e - 39$; hypergeometric test). (B) 24 of the 34 differential dependencies reported by the univariate test to be associated with BRAF(A) or Skin cancer are associated with both the mutation and Skin cancer. In contrast, 0 of the 26 differential dependencies reported by SuperDendrix to be associated with BRAF(A) or Skin cancer is associated with both features. (C) 130 cell lines with KRAS(A) mutation are significantly enriched for Pancreatic cancer (31/130 cell lines with KRAS(A), fold enrichment = 5.4, $P = 2.7e - 24$), Colon cancer (17/130 cell lines with KRAS(A), fold enrichment = 2.8, $P = 2.7e - 6$), Lung cancer (31/130 cell lines with KRAS(A), fold enrichment = 1.7, $P = 0.0002$), and Bile duct cancer (10/130 cell lines with KRAS(A), fold enrichment = 2.3, $P = 0.002$). (D) 14 of the 34 differential dependencies reported by the univariate test to be associated with KRAS(A) or its enriched cancer types are associated with both the mutation and at least one of the enriched cancer types. In contrast, 0 of the 40 differential dependencies reported by SuperDendrix to be associated with KRAS(A) or its enriched cancer types are associated with both the mutation and at least one of the enriched cancer types.

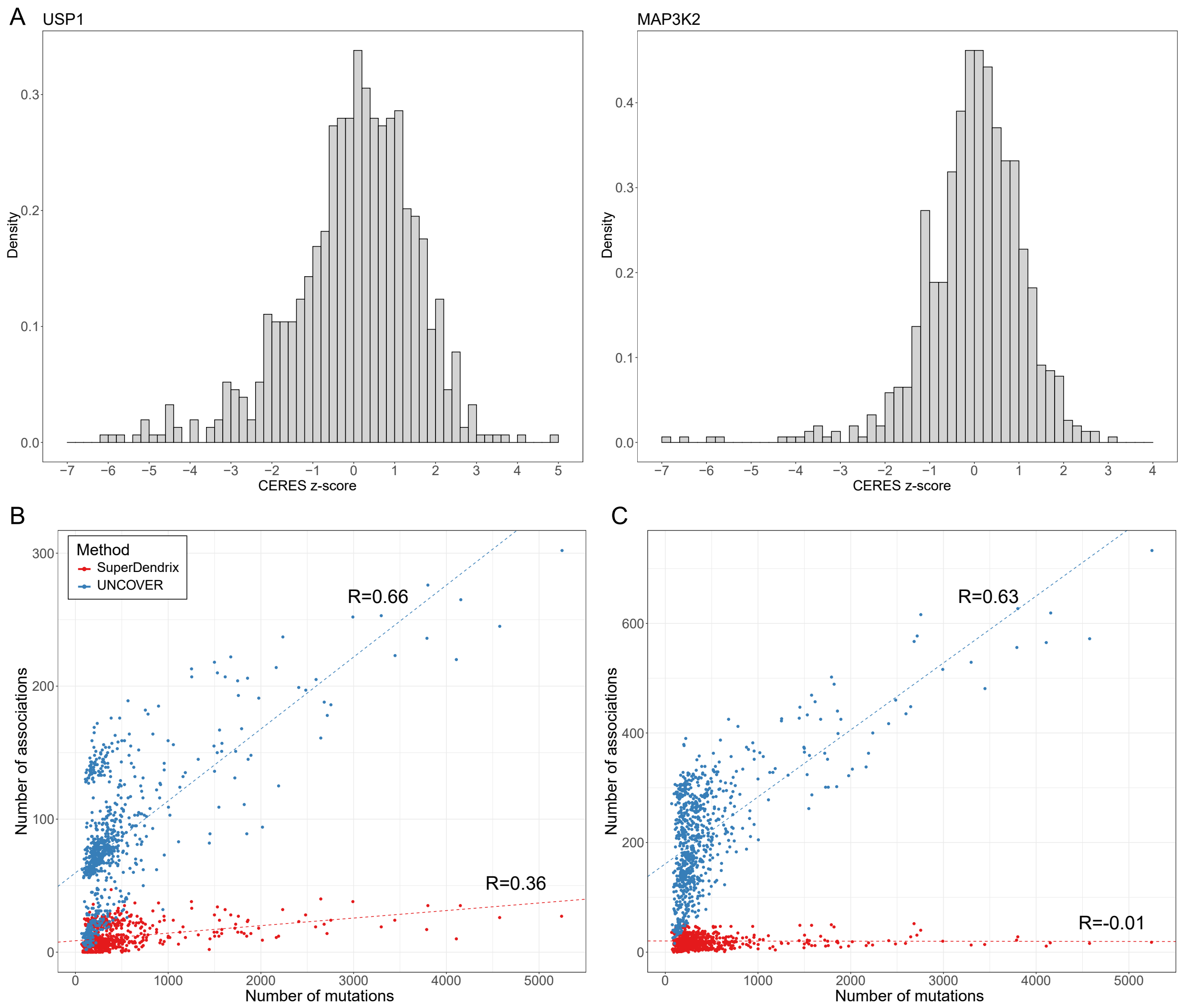

Figure S14: **Associations reported by UNCOVER include potential false positives, Related to STAR Methods.** (A) CERES z-score distributions of *USP1* and *MAP3K2*. UNCOVER identifies associations for USP1 and MAP3K2 whose dependency scores are unimodal and better fit by a single t-distribution than by a mixture of 2 t-distributions. (B-C) Correlation between the number of significant associations in a cell line and the total number of mutations in the cell line. (B) Correlation is lower in SuperDendrix for mutation results (SuperDendrix: $R = 0.36$, UNCOVER: $R = 0.66$). (C) Correlation is lower in SuperDendrix also for cancer type results (SuperDendrix: $R = -0.01$, UNCOVER: $R = 0.63$). Correlation was computed by comparing the number of significant features in a cell line with the number of mutations in the cell line.

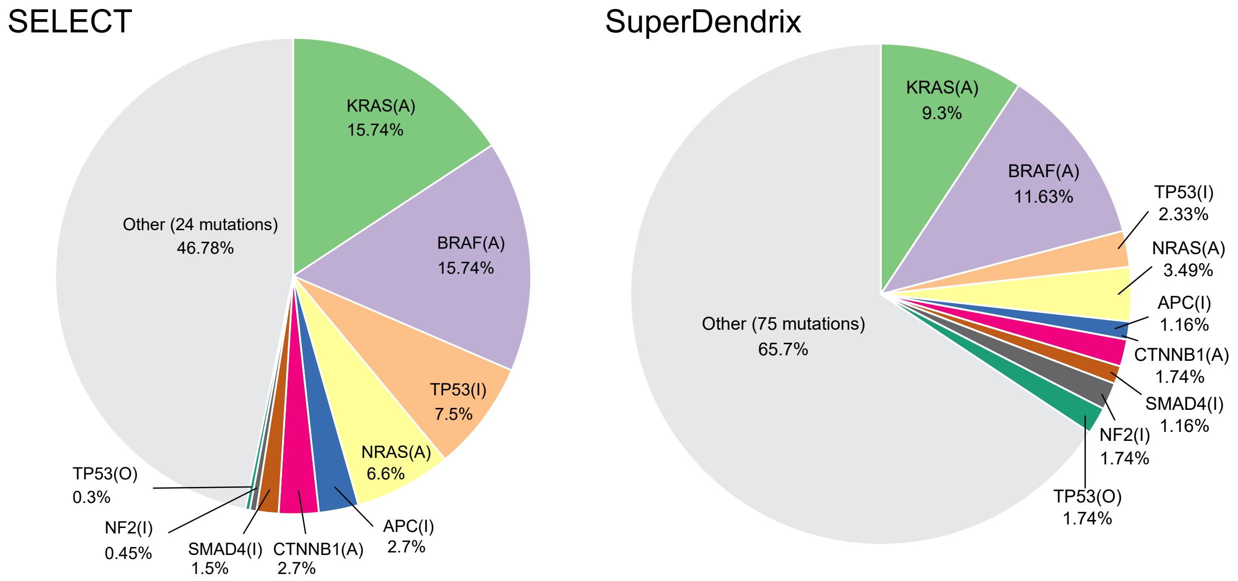

Figure S15: **Associations found using pairs of mutations identified by SELECT are dominated by mutations in a small number of frequently mutated genes, Related to STAR Methods.** SELECT associations include 24 pairs containing a total of 33 mutations, while SuperDendrix identifies associations between 87 sets containing a total of 84 mutations. Associations for 46% of the differential dependencies reported by SELECT to have associated mutations include four frequent mutations, KRAS(A) (130 cell lines), BRAF(A) (65 cell lines), TP53(I) (495 cell lines), or NRAS(A) (48 cell lines), compared to 27% of the differential dependencies reported by SuperDendrix. The percentages indicate the proportion of differential dependencies that are reported to have associated mutations.

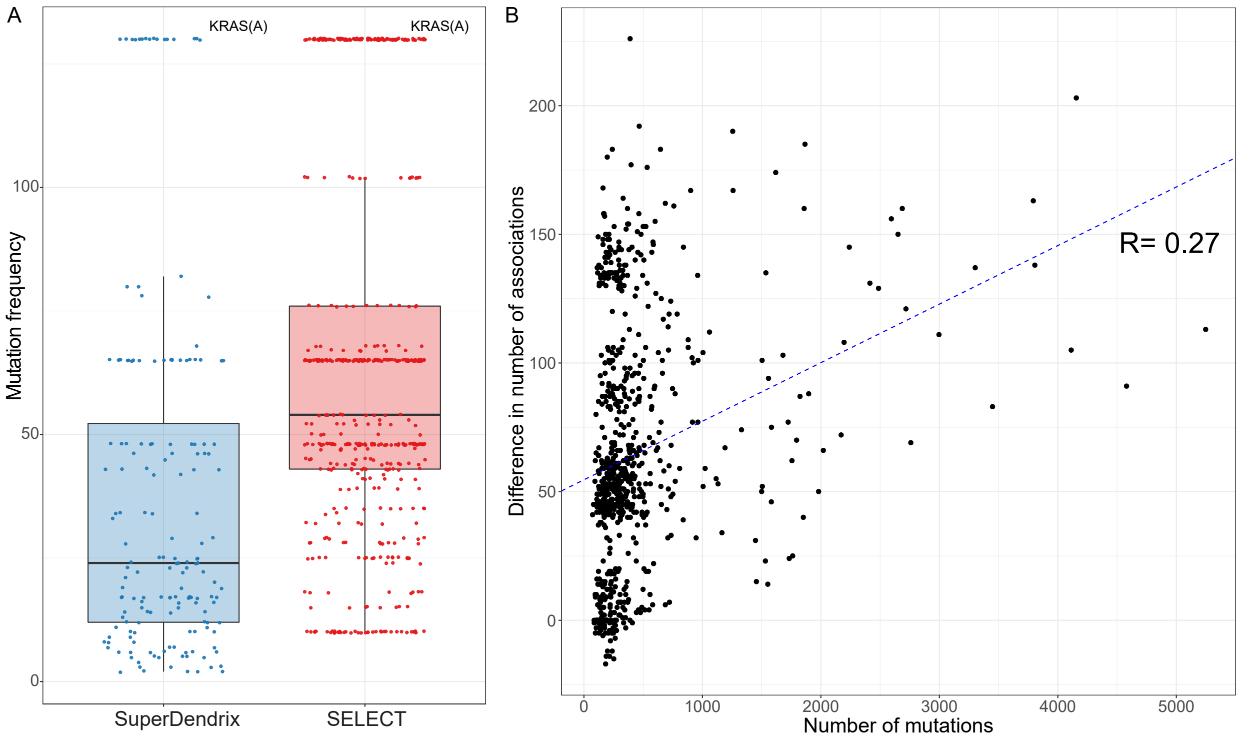

Figure S16: **Associations reported by SELECT are biased towards frequent features and cell lines with high number of mutations, Related to STAR Methods.** (A) Mutations in associations identified by SELECT have higher frequency than those identified by SuperDendrix (SELECT: 64.8, SuperDendrix: 38.6, $P = 1.2e-14$; t-test). Mutation TP53(I) with outlier frequency (495 cell lines) was excluded from comparison. (B) The differences between the number of associations reported by SELECT and SuperDendrix for each cell line is positively correlated with the number of mutations in the cell line ($R = 0.27$, $P = 2.02e-14$; Pearson correlation).
